# From Edge Transformer to IoT Decisions: Offloaded Embeddings for Lightweight Intrusion Detection

**DOI:** 10.3390/s26020356

**Published:** 2026-01-06

**Authors:** Frédéric Adjewa, Moez Esseghir, Leïla Merghem-Boulahia

**Affiliations:** 1 LIST3N, University of Technology of Troyes, 12 rue Marie Curie, 10010 Troyes CEDEX, France; moez.esseghir@utt.fr (M.E.); leila.merghem_boulahia@utt.fr (L.M.-B.); 2 Computer Science Departement, Saint Jean Ingénieur, Yaounde 749, Cameroon

**Keywords:** semantic embeddings, computation offloading, edge computing, BERT, artificial intelligence of things, efficient intrusion detection

## Abstract

The convergence of Artificial Intelligence (AI) and the Internet of Things (IoT) is enabling a new class of intelligent applications. Specifically, Large Language Models (LLMs) are emerging as powerful tools not only for natural language understanding but also for enhancing IoT security. However, the integration of these computationally intensive models into resource-constrained IoT environments presents significant challenges. This paper provides an in-depth examination of how LLMs can be adapted to secure IoT ecosystems. We identify key application areas, discuss major challenges, and propose optimization strategies for resource-limited settings. Our primary contribution is a novel collaborative embeddings offloading mechanism for IoT intrusion detection named SEED (Semantic Embeddings for Efficient Detection). This system leverages a lightweight, fine-tuned BERT model, chosen for its proven contextual and semantic understanding of sequences, to generate rich network embeddings at the edge. A compact neural network deployed on the end-device then queries these embeddings to assess network flow normality. This architecture alleviates the computational burden of running a full transformer on the device while capitalizing on its analytical performance. Our optimized BERT model is reduced by approximately 90% from its original size, now representing approximately 41 MB, suitable for the Edge. The resulting compact neural network is a mere 137 KB, appropriate for the IoT devices. This system achieves 99.9% detection accuracy with an average inference time of under 70 ms on a standard CPU. Finally, the paper discusses the ethical implications of LLM-IoT integration and evaluates the resilience of LLMs in dynamic and adversarial environments.

## 1. Introduction

Kevin Ashton, a pioneer in the field, once remarked: “The Internet of Things has the potential to change the world, just as the internet did.”. This statement has become increasingly relevant as we witness the widespread adoption of connected devices across nearly every domain involving communication between entities, whether machines, humans, or both; And according to trends, the number of connected devices is expected to exceed 40 billion by 2030 (https://iot-analytics.com/number-connected-iot-devices/ (28 October 2025)). This unprecedented growth is reshaping modern life, with transformative impacts across domains such as smart homes, healthcare, transportation, and industrial systems. More specifcally, in smart homes, IoT powered by machine learning in most of the cases allows establishing an adaptive environment that aims to improve the safety, the efficiency and the comfort of the users [1]. The miniaturization of these devices enables body networks and healthcare IoT equipped with sensors to track physiological parameters like heart rate, muscle activity, and electrodermal responses, providing continuous real-time feedback that supports early diagnosis and improved patient care and healthcare services and enables remote health monitoring [2,3].

IoT technologies have also contributed significantly to energy management. Utilizing Wireless Sensor Networks (WSNs) and power line communication, smart grids and IoT-enabled meters allow for the real-time monitoring and optimization of energy consumption [4]. Such systems facilitate efficient demand-response mechanisms and dynamic interactions between users and service providers. Looking at the field of smart cities, IoT is transforming the urban landscapes into intelligent, responsive ecosystems, which enhances the quality of life for citizens; it strengthens public safety through connected surveillance and safeguards citizen well-being by continuously monitoring environemental conditions [3].

However, the proliferation of connected devices also introduces an expansive attack surface that attracts malicious actors. Cybercriminals increasingly exploit vulnerabilities in IoT systems to disrupt operations, compromise user privacy, or exfiltrate sensitive data [5]. Traditional cybersecurity mechanisms often fall short in these environments due to the heterogeneity and resource constraints of IoT devices, leaving them particularly vulnerable to sophisticated threats, including zero-day exploits [6,7].

To address these challenges, artificial intelligence has emerged as a powerful tool for securing IoT systems. Early AI approaches relied on static rule-based methods, which proved inadequate for complex or high-dimensional data. Machine learning techniques introduced adaptability through data-driven learning, and deep learning further extended these capabilities by enabling advanced multimodal data processing through parallel computing architectures.

Recent years have witnessed a surge in the application of artificial intelligence (AI) to cybersecurity [8]. Among the most transformative developments are Large Language Models (LLMs). These models are built upon the Transformer architecture, a class of deep neural networks that relies on the self-attention mechanism [9]. This architecture has achieved state-of-the-art performance in natural language processing due to its ability to capture long-range contextual dependencies in sequential data, significantly outperforming earlier models such as recurrent neural networks (RNNs) and long short-term memory networks (LSTMs).

In network-related tasks, Transformer-based models exhibit strong potential for applications such as log analysis, threat detection, and incident response. However, their effectiveness depends on appropriate data preprocessing, as raw network representations (e.g., PCAP files) are not directly compatible with language models. To address this limitation, network data can be transformed into textual representations using techniques such as Privacy-Preserving Fixed-Length Encoding (PPFLE) combined with byte-pair encoding (BPE) tokenization [10]. These transformations enable the construction of contextual representations over network traffic, rendering the data exploitable by Transformer-based models.

The convergence of AI and the Internet of Things (IoT), commonly referred to as the Artificial Intelligence of Things (AIoT) [11], seeks to leverage AI models, including LLMs, to process the massive volumes of data generated by distributed IoT networks. Within this context, securing IoT infrastructures against cyber threats has become a critical challenge.

In this work, we aim to develop an intrusion detection scheme that leverages the rich representations embedded in network flow data. Specifically, we employ BERT [12], an encoder-only Transformer model that is context-dependent and capable of generating semantically rich embeddings based on token position and surrounding context [13]. BERT is particularly well suited for downstream tasks involving sequential data, while remaining open source, architecturally flexible for fine-tuning, and comparatively lightweight when contrasted with more advanced encoder-only Transformer models. These characteristics enable efficient deployment and improved inference time, which are critical requirements for practical intrusion detection systems. Since these embeddings are inherently task-dependent, we fine-tune the bert-base model to adapt its pretrained knowledge to the network intrusion detection task.

Despite the promising potential of LLMs (which, in the academic context of this work, broadly refer to Transformer-based models) for enhancing cybersecurity, particularly in IoT settings, their deployment in resource-constrained and heterogeneous IoT environments remains insufficiently explored. This gap motivates further investigation into efficient and scalable learning frameworks tailored to such constrained scenarios.

To bridge the existing research gap, this paper makes the following key contributions:

- We provide a structured overview grounded in the fundamentals of LLM-based cybersecurity for IoT systems, addressing the current lack of comprehensive frameworks in this domain.
- We establish the foundational concepts that support IoT security, including typical IoT architectures, their associated vulnerabilities, and the emerging role of Large Language Models in cybersecurity contexts.
- We analyze the evolving threat landscape in IoT environments, identifying common attack vectors and layer-specific vulnerabilities that challenge secure system design and operation.
- We present a detailed examination of LLM applications in IoT cybersecurity, which includes anomaly and intrusion detection, threat intelligence, secure configuration management, and human-in-the-loop security workflows.
- We propose SEED (Semantic Embeddings for Efficient Detection), an approach that leverages Edge-derived Transformer semantics to enable lightweight and accurate intrusion detection on resource-constrained IoT devices. In SEED, a BERT-based Transformer model is trained and deployed at the edge level, where it is reduced by approximately 90% of its original size, resulting in a compact 41 MB model responsible for generating rich semantic embeddings of network traffic. These embeddings are then used to train a highly compact IoT-level neural network of only 137 KB, which achieves up to 99% accuracy for both binary (normal vs. abnormal) and multi-category threat detection tasks. The IoT-level classifier maintains an average inference latency of under 70 ms on CPU hardware, demonstrating the practicality and efficiency of SEED for real-world IoT deployments.
- We illustrate the practical potential of LLMs in IoT cybersecurity through empirical case studies, demonstrating improvements in threat detection accuracy and malware analysis, particularly when hybrid or ensemble learning techniques are employed.
- Finally, we discuss adversarial robustness and ethical implications of integrating LLMs into IoT security systems, emphasizing the need for secure, interpretable, and resilient model deployments, especially under changing or potentially hostile conditions.

## 2. Background and Related Work

This section provides an overview of IoT architectures and their associated security requirements, followed by a discussion of the role of AI in cybersecurity and the recent emergence of LLMs as transformative tools in this domain. It concludes by presenting relevant works that have applied LLMs to various cybersecurity tasks.

### 2.1. IoT Architectures and Security Needs

**Figure 1:**
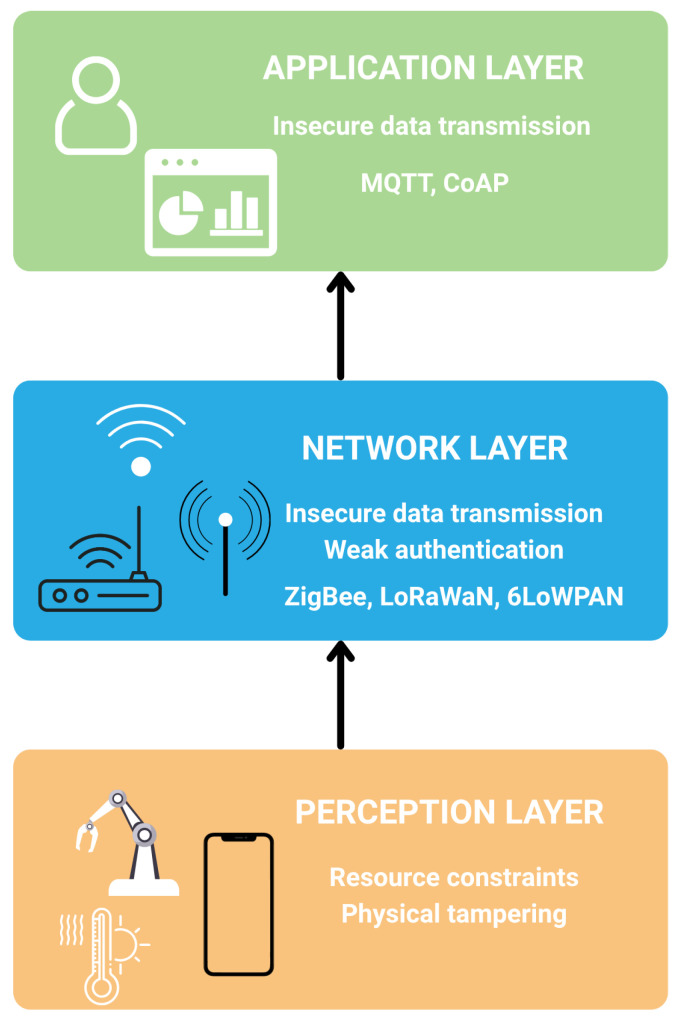
Layered IoT Architecture and Associated Security Challenges.

IoT systems are typically structured into three primary layers: the perception layer, the network layer, and the application layer. Each of these plays a distinct role in enabling data-driven services and presents unique security challenges. Figure 1 illustrates the classical 3 layered IoT architecture with associated security challenges for each layer.

The perception layer consists of sensors, actuators and embedded devices that interact directly with the physical environment. These components are often constrained in terms of power, processing capabilities and memory, making them susceptible to physical tampering and hardware-level attacks. Due to their limited computational capacity, these devices frequently operate without robust encryption or authentication mechanisms, exposing them to risks such as spoofing or side-channel attacks [7].

The network layer facilitates data transmission between devices and cloud or edge infrastructures. It commonly employs lightweight communication protocols such as 6LoWPAN, Thread, Routing protocol for low-power and lossy Networks, Zigbee, LoRaWAN, which are optimized for low-power and lossy networks. While these protocols support efficient communication, they often trade off security for performance, lacking built-in support for strong authentication, data integrity, or end-to-end encryption [5].

The application layer delivers user-facing services and integrates analytics and decision-making functions. This layer must enforce secure access controls, data privacy policies, and trusted interactions with users and third-party systems. However, vulnerabilities can emerge from poorly implemented APIs, weak user authentication schemes, or insecure firmware updates [6]. Table 1 summarizes the security breaches and consequences for the layers of the IoT architecture.

### 2.2. Threats and Attack Surfaces

IoT devices are exposed to a wide range of cyberattacks that exploit their ubiquitous connectivity and limited computational resources. These attacks can be categorized according to the targeted component or layer of the system.

- Physical Attacks: Involve direct manipulation of hardware components, including tampering, jamming, and node capture.
- Network Attacks: Target communication protocols through routing manipulation, sniffing, Man-in-the-Middle (MITM) interception, and DoS/DDoS flooding [14].
- Software Attacks: Exploit firmware or embedded code via malware injection, rootkits, code injection, and privilege escalation.
- Application-Layer Attacks: Focus on user-facing services, including XSS, SQL injection, and phishing.
- Authentication and Authorization Attacks: Aim to bypass access controls using brute force, password cracking, and replay techniques.

### 2.3. Artificial Intelligence for Cybersecurity

AI has increasingly become integral to cybersecurity, offering scalable solutions to detect, predict, and respond to threats in real time. Traditional rule-based systems, while useful for known threats, often struggle to adapt to the dynamic and complex nature of modern attack vectors. Machine learning techniques, particularly those that leverage large volumes of behavioral and system data, provide more adaptive and context-aware mechanisms for security enforcement.

Two primary learning paradigms are employed in AI-driven cybersecurity: supervised learning and unsupervised learning. Supervised learning relies on labeled datasets to train models capable of classifying known threats, such as in malware or phishing detection. In contrast, unsupervised learning does not require labeled data and is commonly used for anomaly detection, enabling systems to identify unusual patterns that may signify zero-day attacks or insider threats [8].

Despite their promise, deep learning (DL) and other advanced ML techniques face several limitations in cybersecurity contexts. Notably, DL models often require large volumes of high-quality, labeled data, which are difficult to obtain in security settings due to privacy constraints and the rarity of certain attack types. Furthermore, these models are susceptible to model drift, where changes in attacker behavior or system configurations degrade the model’s performance over time, necessitating frequent retraining and monitoring.

AI-powered approaches have found widespread use in applications such as malware classification, phishing detection, and network anomaly detection. These systems analyze static and dynamic features of executables, email contents, and traffic patterns to infer malicious intent. However, the effectiveness of such models depends heavily on their robustness to adversarial manipulation and the relevance of training data to evolving threat landscapes [8].

### 2.4. LLM for Security and IoT

The proliferation of connected devices has revolutionized various sectors, enabling a broad range of applications. Among the most prominent examples are voice assistants such as Apple’s Siri and Amazon’s Alexa. Initially, these systems offered limited user-friendliness due to their robotic and unnatural responses. The integration of LLMs has significantly transformed human–machine interaction. Owing to their advanced natural language understanding and generation capabilities, LLMs enable more fluid, context-aware, and human-like conversations. As a result, user experiences have improved significantly.

Beyond conversational agents, the integration of AI and IoT has enabled intelligent, autonomous decision-making across diverse domains. Traditional IoT systems relied heavily on expert-defined rule sets, which, although functional, lacked scalability and adaptability. With the advent of LLMs, this paradigm has shifted. In smart manufacturing, for instance, LLM-empowered IoT systems can analyze sensor data in real time to optimize production lines, detect anomalies, and recommend precise adjustments to enhance efficiency and resource utilization.

Modern IoT devices are equipped with advanced sensors that generate vast quantities of heterogeneous data. This data fuels applications ranging from gaze tracking using Inertial Measurement Units (IMUs) [15] to depth estimation for XR, VR, and robotics. Systems like RGBDGaze incorporate RGBD data for enhanced gaze estimation accuracy. Furthermore, embedded cameras on drones or satellites enable 3D reconstruction of real-world environments, crucial for applications in mapping and environmental monitoring.

Processing such complex and substantial data requires sophisticated computational models. LLMs, especially in their multimodal variants, address this challenge by effectively interpreting and correlating diverse data modalities, including text, images, audio, and video [15]. This capability significantly enhances IoT applications, enabling more context-aware, adaptive, and intelligent system behaviors.

In the field of education, LLMs and generative AI also offer solutions to the longstanding challenge of personalized learning [16]. When combined with IoT-based student monitoring systems, these models can dynamically generate educational content tailored to individual cognitive and cultural profiles. Such AI-driven IoT frameworks offer promising tools to reduce learning gaps and improve pedagogical outcomes.

### 2.5. Related Work

To assess the practical viability of LLMs in IoT security, this section highlights key studies demonstrating their effectiveness across various threat scenarios. Ref. [10] introduced SecurityBERT, an LLM-based threat detection system for IoT and IIoT environments. Using privacy-preserving fixed-length encoding (PPFLE) to preprocess traffic data, it achieved a 98.2% detection accuracy with sub-150 ms inference latency on an average CPU. Its compact size (16.7 MB) makes it suitable for deployment on edge devices. Similarly, ref. [17] employed fine-tuned LLMs to achieve 94.9% accuracy in DDoS detection, surpassing traditional MLPs in few-shot learning settings. These findings illustrate the capacity of LLMs to detect sophisticated threats in resource-constrained environments. Ref. [18] evaluated BERT and GPT-2 for malware detection using datasets such as TON-IoT [19] and NSL-KDD [20]. BERT consistently outperformed GPT-2, achieving up to 94.0% accuracy on TON-IoT due to its encoder-only architecture, which enables more effective context modeling. However, practical deployment challenges on IoT hardware were not addressed, indicating the need for further work on model optimization.

In another study, ref. [6] proposed a hybrid architecture combining GPT-4 with autoencoders for enhanced preprocessing and interpretability. The LLM component recommends preprocessing strategies and generates explanatory insights post-detection. Although the model showed improved detection accuracy, the authors did not evaluate its deployability on actual IoT devices. Ref. [21] explored an ensemble approach combining BART for packet prediction and BERT for accuracy assessment, using the CICIoT2023 dataset [22]. BART achieved 98.26% classification accuracy, while BERT provided robust contextual validation. However, the study lacks a detailed analysis of training cost and inference latency, factors critical for real-world IoT applications.

In our prior work [23], we applied federated learning with a lightweight BERT variant for intrusion detection on IoT devices. Unlike centralized learning approaches, our method trains models locally and aggregates parameters globally to preserve data privacy. Although parameter transfer (1.17 million parameters) introduced delays, reducing the model depth significantly improved responsiveness, making the approach feasible for edge deployments.

A prevalent shortcoming in the existing literature is the insufficient consideration of the resource-constrained nature of IoT devices, which are typically characterized by limited battery life and storage capacity. This limitation necessitates the development of solutions that are not only accurate but also highly efficient in terms of energy and memory utilization. To bridge this gap, we propose a novel approach that offloads the computationally intensive feature learning phase from the constrained devices, as summarized in Table 2. Our methodology leverages the superior representational capacity of a transformer model, which is trained in a resource-rich environment. The acquired knowledge is then transfer into a compact Feed-Forward Neural Network (FFNN) deployed on the IoT device. This strategy achieves an optimal balance between high detection performance and minimal resource consumption.

## 3. Applications of LLMs for IoT Cybersecurity

LLMs, with their advanced reasoning and natural language capabilities, are increasingly used to address the cybersecurity challenges of the IoT. This section explores the major application areas of LLMs in IoT security, including threat detection, log analysis, secure configuration, and interactive support for human operators.

### 3.1. Anomaly and Intrusion Detection

LLMs have demonstrated strong potential in detecting abnormal behaviors in network traffics. Models such as BERT and GPT-2 have been applied to analyze network traffic for identifying deviations from baseline behavior [18]. For instance, ref. [10] developed SecurityBERT, a lightweight model with 98.2% detection accuracy and sub-150ms inference time, suitable for edge environments.

Hybrid approaches combining LLMs with autoencoders have also been proposed. Ref. [6] used GPT-4 to guide anomaly detection and recommend preprocessing strategies, improving explainability and detection accuracy. Ensemble architectures, such as those by [21], integrate BART for packet prediction with BERT for validation, achieving over 98% accuracy on CICIoT2023 dataset.

### 3.2. Threat Intelligence and Log Analysis

One of the most promising use cases for LLMs in IoT cybersecurity is the automated extraction and summarization of cyber threat intelligence. Fine-tuned on structured datasets like MITRE ATT&CK or CAPEC, LLMs can produce comprehensive threat reports, interpret attack patterns, and generate context-aware incident descriptions [24]. These outputs help improve situational awareness and assist in identifying emerging attack trends.

Furthermore, LLMs are increasingly used to parse and interpret large-scale log files, correlating events across time and systems. Their contextual understanding enables them to generate incident summaries that are both technically accurate and readable by human analysts.

### 3.3. Secure Configuration and Policy Generation

LLMs also contribute to identifying misconfigurations and suggesting secure alternatives in IoT environments. They can detect vulnerabilities in configuration scripts, code snippets, and deployment templates, especially those obfuscated to evade static analysis [6,8]. Emerging models like SecureFalcon [25] improve interpretability and reduce false positives during automated security audits.

Moreover, LLMs can be fine-tuned to generate security policies or hardening guides tailored to specific IoT platforms or device constraints, enabling scalable and proactive security design.

### 3.4. Human-in-the-Loop: Security Assistants and ChatOps

Beyond automated analysis, LLMs serve as natural language-based assistants that support human analysts in real-time decision-making. Their ability to explain security alerts in plain language, provide incident summaries, and recommend responsive actions allows for faster and more informed interventions [6].

This aligns with the emerging practice of ChatOps, where human analysts interact with automated systems via chat-based interfaces. LLMs embedded into such workflows enable collaborative threat investigation, knowledge base querying, and real-time documentation generation, bridging the gap between automation and human expertise.

## 4. SEED Framework

In this section, we present our proposed SEED scheme, which leverages the computational power of BERT models at the edge level to train a compact neural network deployed at the IoT level for efficient traffic prediction and anomaly detection.

### 4.1. System Overview

**Figure 2:**
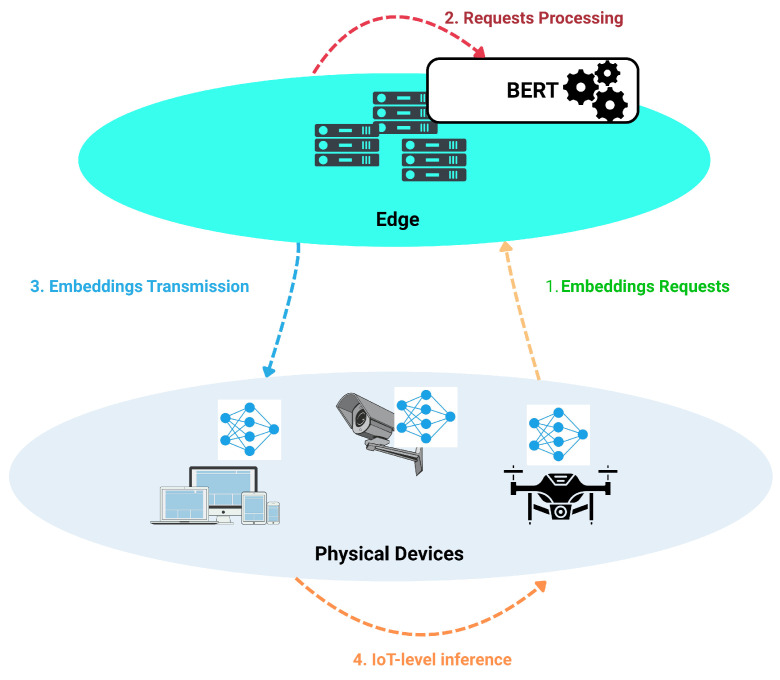
Overview of the SEED architecture, where a compressed edge-level Transformer model generates semantic embeddings that are offloaded to a lightweight IoT-level classifier for efficient intrusion detection.

This subsection provides a conceptual overview of the SEED architecture presented in Figure 2, highlighting the interaction between the edge-based BERT model and the resource-constrained IoT devices.

As discussed previously, Transformer-based models such as BERT have demonstrated outstanding performance in diverse tasks involving sequential data processing [10,21]. However, their high computational and memory requirements render their direct deployment on IoT devices impractical, as such environments typically lack the necessary resources (e.g., GPUs) for large-scale model training and inference.

To address these limitations, we propose a collaborative Edge-to-IoT learning scheme, referred to as SEED, in which a Transformer-based model is trained at the edge layer, while a compact neural network is deployed at the IoT layer. Compared to IoT devices, edge nodes offer significantly greater computational resources, enabling the training of a BERT-based intrusion detection model capable of learning rich and generalized representations of network traffic patterns across multiple attack types.

Since networks generate unstructured and vast amoung of data that make it difficult to extract direct insights [26], the edge-level model encodes network traffic in a high-dimensional embedding space, thus capturing complex semantic relationships between packet-level features. As stated earlier, raw network data are not directly exploitable by transformer-based models. To overcome this limitation, Algorithm 1 converts the network dataset into a textual representation by concatenating each feature name with its corresponding feature value, separated by a predefined marker (“$” in our case). This process is applied uniformly across the entire dataset.
**Algorithm 1** Data preprocessing: feature-wise textual serialization**Input:** Dataset $D={\left\{\left(x_{i},y_{i}\right)\right\}}_{i=1}^{N}$
**Input:** Feature set F                ▹ selected features to encode**Input:** Hash function H(·)        ▹ privacy-preserving fixed-length encoder**Output:** Serialized corpus $S={\left\{s_{i}\right\}}_{i=1}^{N}$
1:  Initialize S←[]2:  **for** $(x_{i},y_{i})\in D$ **do**3:      Initialize token list $T_{i}\leftarrow \left[\;\right]$4:      **for** f∈F **do**5:          $v\leftarrow x_{i}\left[f\right]$               ▹ value of feature *f* in sample $x_{i}$6:          $t\leftarrow H\;\left(f\;∥\;\$\;∥\;\mathrm{toString}(v)\right)$7:          Append *t* to $T_{i}$8:      **end for**9:       $s_{i}\leftarrow \mathrm{join}\left(T_{i},\;””\right)$             ▹ space-separated token sequence10:     Append $s_{i}$ to S11:**end for**12:**return** S

Once the textual corpus is constructed, a new vocabulary of size 32,522 (corresponding to the default BERT vocabulary size) is learned from scratch based on this newly formed context. Consequently, the model is trained exclusively on this domain-specific vocabulary, allowing it to learn deep semantic representations tailored to network traffic data. Unlike the approach in [27], our method does not rely on dataset matching. Instead, the proposed preprocessing strategy can be applied to multiple heterogeneous datasets (e.g., IoT traffic and traditional network traffic), enabling the construction of a broader contextual space to enable a more flexible and generalizable intrusion detection system.

The key idea is to exploit these embeddings to bridge the performance–efficiency gap between edge and IoT. Once trained, the edge model generates embeddings for incoming IoT traffic and transmits them to the IoT level, where a lightweight neural network processes the embedding vectors instead of raw packet features. In our framework, we use the condensed representation produced by BERT as the embedding of each network traffic sample (processed as batch of size $B_{size}$), specifically the hidden state associated with the [CLS] token from the final encoder layer due to its compactness. Our confirmation outputs an embedding vector with a fixed-length of dimension 256, which serves as a global, context-aware representation of the entire input sequence. The inference performed by the IoT-side model classifies traffic instances as *benign* or *anomalous* and even performs fine-grained classification, enabling near real-time detection with minimal computational overhead.

The overall workflow is summarized by two core processes. Algorithm 2 depicts the training process of the BERT model at the edge level. In this phase, the model learns to classify various types of attacks while concurrently learning to represent network flows in a high-dimensional space. Algorithm 3 outlines the lightweight model inference at the IoT level. Here, instead of processing raw network flows directly, which can be computationally intensive due to the feature learning phase, the model requests the embedding representation of these flows from the EdgeBERT model. It then trains a Feed-Forward Neural Network (FFNN) on these rich semantic embeddings. Finally, the model classifies traffic as anomalous by comparing the output probability of the classification head with a threshold τ (typically set to 0.5 for binary classification).
**Algorithm 2** Edge-Level BERT Model Training and Embedding Generation**Input:** Dataset $D={\left\{\left(x_{i},y_{i}\right)\right\}}_{i=1}^{N}$ of network traffic samples**Output:** Trained BERT model $M_{BERT}$1:  Initialize BERT-base model $M_{BERT}$ with pre-trained weights2:  **for** each mini-batch $\left(x_{b},y_{b}\right)$ in *D* **do**3:       $x_{b}$ into input sequence $s_{b}$             ▹ Tokenize and encode4:       $h_{b}=M_{BERT}\left(s_{b}\right)$             ▹ Compute hidden representations5:       ${\hat{y}}_{b}=f\left(h_{b}\right)$                 ▹ Compute classification output6:       $L=\mathrm{CrossEntropy}({\hat{y}}_{b},y_{b})$               ▹ Compute the loss7:       Update $M_{BERT}$ parameters via backpropagation8:  **end for**9:  Freeze the trained model parameters10:**return** $M_{BERT}$

**Algorithm 3** IoT-Level Lightweight Neural Network Inference**Input:** Compact neural network $M_{IoT}$**Input:** Incoming IoT traffic sample $x_{i}$**Output:** Predicted class label ${\hat{y}}_{i}\in \left[0,1\right]$
1:$e_{i}=M_{BERT}\left(x_{i}\right)$       ▹ Embeddings request, processing and transmission2:${\hat{y}}_{i}=M_{IoT}\left(e_{i}\right)$                 ▹ Compute prediction score3:**if** ${\hat{y}}_{i}\geq \tau$ **then**4:    Classify traffic as *anomalous*5:
**else**
6:    Classify traffic as *benign*7:
**end if**
8:**return** ${\hat{y}}_{i}$


### 4.2. Compression in SEED

Given the large size and computational demands of LLMs, compression techniques are vital to making them compatible with IoT constraints. These techniques reduce memory usage and inference latency while maintaining acceptable performance.

Quantization

Quantization involves reducing the numerical precision of model weights, which are typically represented in 32-bit floating-point (FP32) format. By converting weights to lower-precision formats such as 8-bit integers (int8) or 4-bit (int4), quantization can reduce model size by a factor of four to eight [11]. This results in lower memory and energy consumption during inference. However, this compression may introduce minor degradation in model accuracy due to reduced precision, particularly in sensitive layers.

Pruning

Pruning techniques eliminate redundant or non-critical parameters from the neural network. These methods exploit the observation that not all weights contribute equally to model performance. By removing near-zero or low-impact weights, pruning reduces both model size and computational burden. However, as this process alters the model’s structure, fine-tuning is typically required post-pruning to recover potential performance loss and ensure inference stability.

Knowledge Distillation

Knowledge distillation transfers knowledge from a large, high-performing teacher model to a smaller, efficient student model [28]. The student learns to replicate the behavior of the teacher, retaining much of the original performance while being significantly smaller and faster. This approach is particularly suitable for resource-constrained devices that cannot accommodate full-scale LLMs.

### 4.3. On-Device and Edge Deployment Strategy

**Figure 3:**
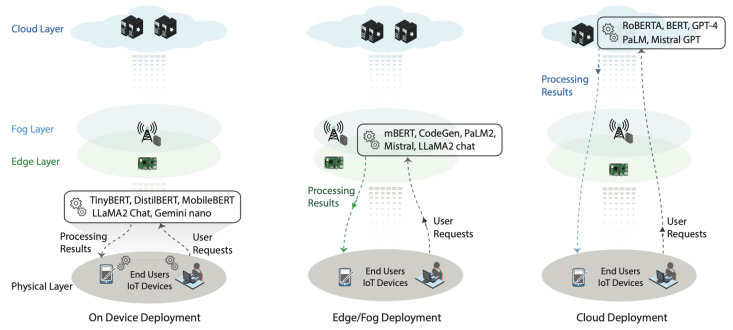
Deployment strategies for LLMs in IoT environments [29].

Beyond model compression, architectural innovations have been introduced to accommodate LLM deployment in constrained edge environments. Researchers have proposed several strategies to minimize latency, optimize resource use, and preserve operational privacy, as illustrated in Figure 3.

Edge Offloading

Edge computing offers a promising paradigm wherein computational tasks are partially or fully offloaded from IoT endpoints to nearby edge servers. Instead of executing LLM inference directly on the device, IoT systems can send requests to more capable nodes, which process the queries and return results [29]. This approach significantly reduces on-device load but introduces concerns around network latency and availability.

In [30], the authors proposed Edge-LLM, a framework for efficient edge-based LLM deployment. It introduces a Layer-wise Unified Compression (LUC) technique to apply layer-specific pruning and quantization. Furthermore, an Adaptive Layer Tuning and Voting scheme reduces memory demands during backpropagation. Coupled with an efficient hardware scheduler, this framework achieved a 2.92× speedup and 4× memory reduction with negligible performance loss, demonstrating its feasibility for on-edge LLM deployment.

Edge-Based and Hybrid Architectures

When edge devices are unable to support inference entirely, edge-based or hybrid solutions are essential. In hybrid models, certain inference stages are performed at the edge, while computationally intensive tasks are handled in the cloud. For instance, ref. [31] proposed a collaborative edge-cloud model in which edge devices generate tokens serially, while cloud systems handle token generation in parallel. This architecture reduces total response time while balancing the trade-offs between performance, latency, and resource use.

Lightweight LLM Variants

An alternative strategy involves using optimized lightweight versions of standard LLMs. Models such as TinyBERT, DistilBERT, and MobileBERT retain core capabilities while drastically reducing parameter count. For instance, TinyBERT achieves up to 96% of BERT’s performance while being 7× smaller and 9× faster. Similarly, recent variants like Gemini Nano and GPT-2 Small are tailored for on-device deployment with acceptable trade-offs in accuracy [29].

Caching and Efficient Inference

In [32], authors covered strategies such as edge LLM caching, gradient checkpointing, and attention optimization, which are essential for deploying LLMs in low-latency, privacy-sensitive applications. The authors also identify key use cases including robotics, healthcare, and smart cities, that benefit from edge-based LLM deployment.

### 4.4. Privacy-Preserving Design

IoT devices frequently process sensitive personal, environmental, and behavioral data. As such, the integration of LLMs must address privacy and data security concerns. Decentralized learning frameworks and secure update mechanisms offer viable solutions.

Federated Learning

Federated Learning (FL) is a decentralized training paradigm that allows models to be trained directly on IoT devices. Instead of transmitting raw data, devices only send model updates to a central aggregator [23,33]. This approach reduces privacy risks by keeping sensitive data local. In the context of LLMs, FL enables continual fine-tuning of models on-device while benefiting from global knowledge sharing.

Secure Model Updates

To enhance the privacy guarantees of FL, additional safeguards such as differential privacy and homomorphic encryption can be applied. Differential privacy introduces calibrated noise into model updates to prevent reverse-engineering of individual data points. Homomorphic encryption enables computation on encrypted data, ensuring that no sensitive information is exposed during training or inference.

Although these strategies significantly improve the feasibility of deploying LLMs in IoT settings, continuous research is essential to address remaining limitations. These include reducing fine-tuning overhead, handling variable network conditions in hybrid models, and developing new architectures that are natively optimized for edge intelligence.

## 5. Experimental Evaluation

### 5.1. Experimental Setup and Evaluation Metrics

To evaluate the effectiveness of our proposed SEED approach, we utilized the widely adopted Edge-IIoTset dataset [34], which contains traffic data from IoT and IIoT systems, including 14 distinct attack types along with normal traffic, yielding 15 classes in total.

We designed three evaluation scenarios:

1. Binary classification scenario: Distinguishes between benign and anomalous traffic.
2. Six-category scenario: Groups attacks into six intermediate categories (five attacks + Normal traffic).
3. Fifteen-class scenario: Performs fine-grained classification across all 14 attack types plus normal traffic.

The first two scenarios (binary and six-category) are intended for execution at the IoT level, leveraging the compact neural network, while the fifteen-class scenario is handled by the large edge-based model due to its higher computational requirements.

**Figure 4:**
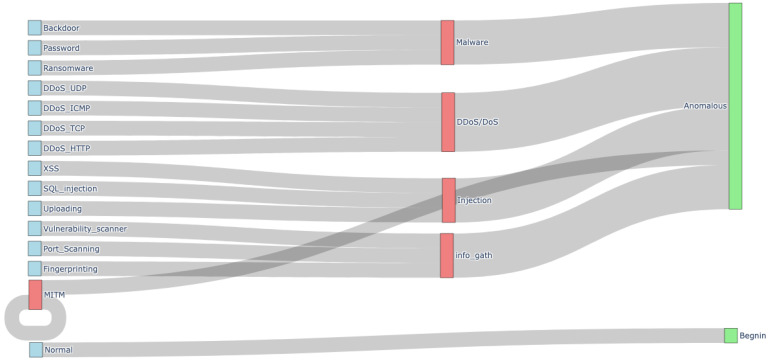
EdgeIIoTset Network Attacks Taxonomy.

Figure 4 illustrates the hierarchical mapping of attack techniques included in the dataset to broader threat categories and finally to anomaly classifications. The visualization demonstrates how individual attacks are grouped into intermediate categories, which are then aggregated into the final classification outputs.

The sive-category scenario is particularly relevant at the IoT level because it captures the most critical IoT threats while remaining computationally efficient for edge devices. This approach balances fine-grained detection with the limited memory and processing capabilities of IoT hardware, providing comprehensive coverage of major IoT threat vectors without overwhelming resource-constrained devices.

Table 3 summarizes the configurations of our SEED models. The EdgeBERT model consists of 4 encoder layers with 256 hidden units, 512 intermediate units, 4 attention heads, and 2 fully connected layers, resulting in over 10 million parameters [23]. The IoT-level classifiers are compact, consisting of two fully connected layers with batch normalization and dropout; the binary and five-category models contain only 33k and 34k parameters, respectively, making them suitable for deployment on resource-constrained IoT devices.

For the Edge-Level model training, we utilized a high-performance server equipped with an NVIDIA A100 GPU with 80 GB of memory, 12 CPU cores, and a total computing power of 312 TFLOPs. To accelerate experiments related to IoT-level model training, we employed an NVIDIA T4 GPU with 16 GB of RAM, 4 CPU cores, and a peak performance of 65 TFLOPs. It is worth noting that the T4 was used solely to speed up training; the IoT models are lightweight enough that they can also be trained on a standard CPU, with longer training times.

To evaluate the performance of our SEED models, we used standard classification metrics including accuracy, precision, recall, and F1-score. These metrics are defined as follows:

- Accuracy measures the proportion of correctly classified instances among all samples:
  (1) $$\mathrm{Accuracy}=\frac{TP+TN}{TP+TN+FP+FN}$$
- Precision quantifies the proportion of correctly predicted positive instances among all instances predicted as positive:
  (2) $$\mathrm{Precision}=\frac{TP}{TP+FP}$$
- Recall measures the proportion of correctly predicted positive instances among all actual positive instances:
  (3) $$\mathrm{Recall}=\frac{TP}{TP+FN}$$
- F1-score is the harmonic mean of precision and recall, providing a balanced metric for classification performance:
  (4) $$F1\text{-}\mathrm{score}=2\;\times \;\frac{\mathrm{Precision}\;\times \;\mathrm{Recall}}{\mathrm{Precision}+\mathrm{Recall}}$$

Here, TP, TN, FP, and FN represent the number of true positives, true negatives, false positives, and false negatives, respectively.

### 5.2. Results and Discussion

The evaluation begins with assessing the performance of the EdgeBERT model. It was trained on the entire training set of the Edge-IIoTset dataset for approximately 15 min on an NVIDIA A100 GPU, corresponding to one epoch. For comparison, training the full BERT-base model on the same dataset would have required an estimated 3.5 h per epoch. After training, EdgeBERT achieved nearly 100% accuracy.

**Figure 5:**
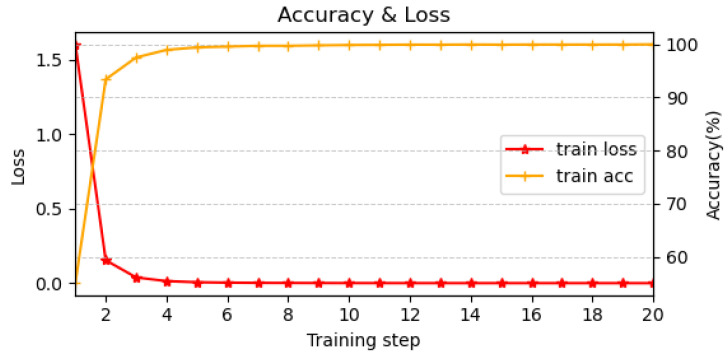
Loss and Accuracy variation of EdgeBERT every 1000 mini batches.

Figure 5 shows the variation of loss and accuracy every 1000 mini-batches during training. Due to the density of the dataset, the model converged rapidly, with convergence effectively reached around the 4000th mini-batch. This fast convergence eliminated the need for additional epochs. Considering both training time and performance, EdgeBERT represents an effective trade-off, as the full BERT model with over 100 million parameters is not strictly necessary for intrusion detection in this context.

**Figure 6:**
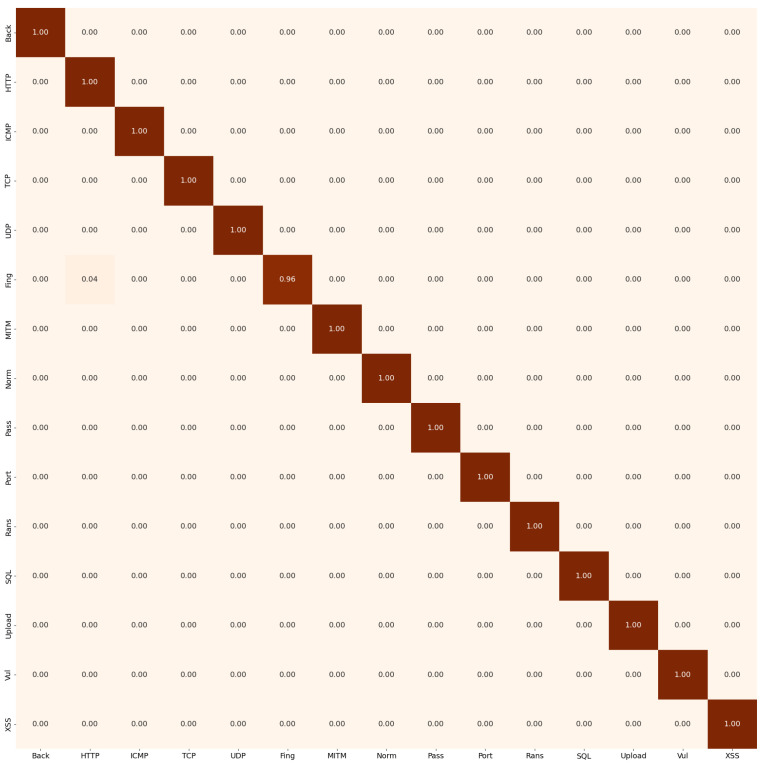
EdgeBERT confusion matrix on 15 classes.

Figure 6 illustrates the confusion matrix for the 15-class classification scenario. The model is able to almost perfectly distinguish between all classes, although a small amount of confusion is observed between the ‘Fingerprinting’ and ‘DDoS-HTTP’ classes.

Table 4 presents the classification report for the Edge-level model. These results highlight the effectiveness of the transformer-based architecture in distinguishing and classifying a broad range of attack types. However, the Fingerprinting class exhibits the lowest performance, with an F1-score of 0.9673. This behavior is partly explained by its relatively small number of instances, which biases the model toward predicting the most semantically similar classes.

Interestingly, other minority classes such as MITM achieve strong performance despite their limited support. This indicates that class imbalance is not the only contributing factor; the semantic separability between classes also plays a crucial role. When two attack types share similar structural or behavioral patterns, the embedding space becomes less discriminative, making fine-grained separation more challenging. In the case of Fingerprinting, its proximity to certain DDoS-related patterns likely contributes to the observed confusion.

At the IoT level, the compact 137 KB neural network achieves an accuracy of 99.99% by following the process outlined in Algorithms 2 and 3. During inference, the IoT model queries the edge-based model to obtain embedding vectors, which are then used as input features for classification. These embeddings are rich and high-dimensional, capturing the semantic characteristics of network traffic, which enables the IoT model to effectively discriminate between different traffic categories.

This level of abstraction is particularly suitable for resource-constrained IoT devices. By relying on precomputed embeddings from the edge, the IoT model eliminates the need for local feature extraction and other computationally intensive operations required for fine-grained classification, while still maintaining near-perfect accuracy.

**Figure 7:**
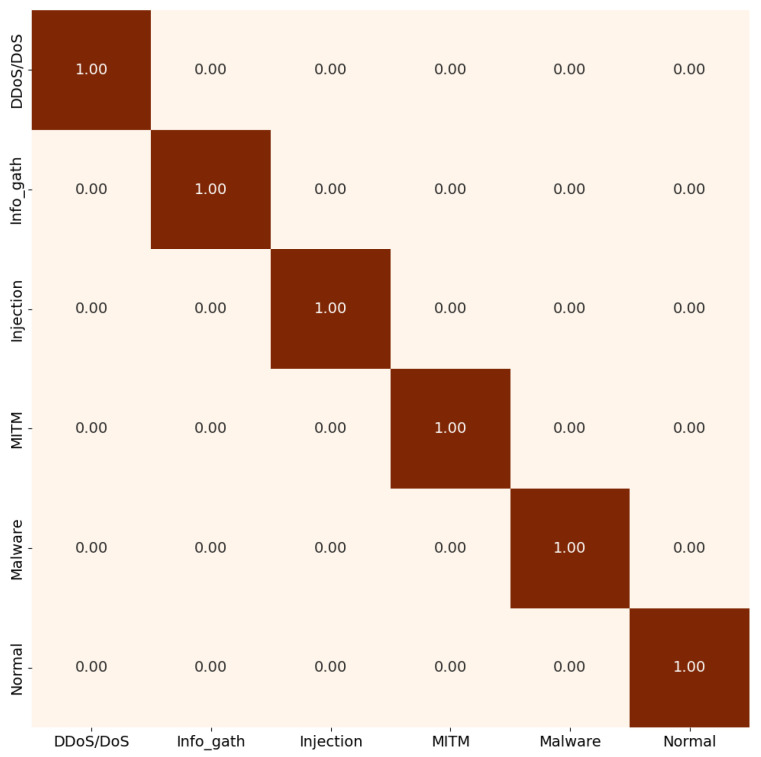
IoT level 5-attacks classification scenario.

Figure 7 illustrates the model’s performance across the five-category scenario. Training the IoT model required approximately 20 min per epoch on a CPU, and convergence was achieved after only two epochs. This demonstrates the efficiency and feasibility of deploying EdgeBERT-derived embeddings on lightweight IoT models, achieving both high accuracy and low computational overhead.

An important consideration in this setup is the network data rate, which directly impacts inference latency. On average, the edge-based model generates embedding batches in 9 ms, while the IoT-level model performs classification in 70 ms on a 2.5 GHz CPU with 4 cores. Table 5 summarizes these metrics. These latency magnitudes align well with real-world IoT requirements, ensuring rapid decision-making and responsiveness. However, a potential bottleneck in this pipeline is the network data rate, which is crucial for transferring the generated embeddings from the edge to the IoT devices. Insufficient throughput or unstable connectivity may increase transmission delays, limiting how quickly the IoT-level model can perform inference.

**Figure 8:**
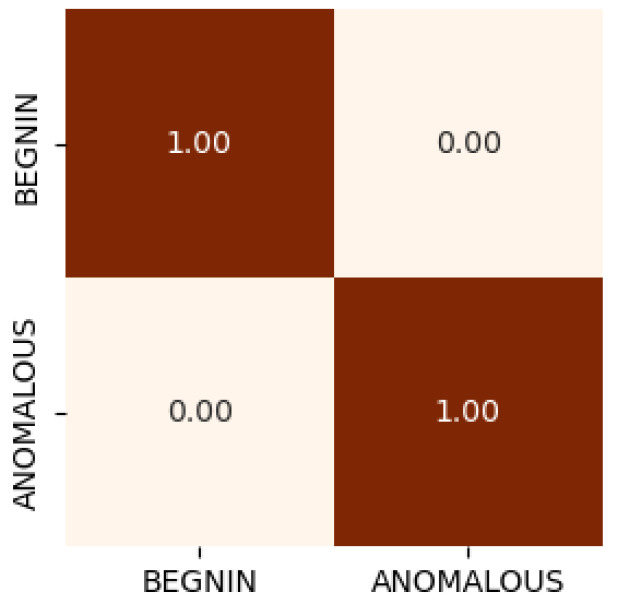
IoT binary classification scenario.

As shown in Figure 8, the IoT-level binary classifier further demonstrates the robustness of our scheme by accurately flagging anomalous traffic instances. This confirms our proposed SEED framework is both computationally efficient and effective for real-time intrusion detection at the network edge.

While promising, the proposed method also presents several limitations that must be addressed for real-world deployment. A primary concern lies in the transmission of embeddings from the edge to IoT devices. Each embedding vector consists of 256 FP32 values (approximately 1 KB), which is lightweight in storage but may impose non-negligible communication demands, especially under constrained or unstable network conditions. Since real-world IoT networks are prone to latency, interference, and bandwidth fluctuations, maintaining continuous embedding transfers may become a performance bottleneck. Future work should therefore investigate mitigation strategies such as local caching, temporary buffering of embeddings, or adaptive communication schedules.

Another important consideration relates to the communication strategy between IoT devices and the edge, particularly during Step 2 of the SEED pipeline illustrated in Figure 2. In our experimental setup, embeddings are requested in batches of size B during inference to optimize throughput. However, determining the optimal batch size and more generally, when communication should be initiated, remains a non-trivial design choice. Excessively frequent communication may overload the network, whereas overly large batches may introduce latency. Exploring dynamic batching or event-triggered communication policies represents a promising direction for improving system efficiency.

Finally, the embeddings transmitted over the network may be susceptible to interception or reverse engineering, potentially revealing sensitive device metadata such as IP addresses or traffic characteristics. Because many IoT communication protocols are lightweight and lack robust built-in security mechanisms, incorporating additional protection layers (e.g., encryption, secure channels, or embedding obfuscation) is essential to preserve confidentiality and prevent data leakage. Addressing these challenges will be key to ensuring a secure and reliable deployment of the SEED framework in practical IoT environments.

It is also crucial to account for network reliability. Designing fallback mechanisms or jump strategies can help ensure continuous service availability, mitigating inference delays or failures caused by intermittent connectivity. Such measures are fundamental to maintaining robust and dependable intrusion detection in practical IoT deployments.

The above results demonstrate the viability of our method, though there remains significant room for improvement, as discussed in the results analysis. With the integration of these enhancements, particularly advancements in the communication strategy between the two-tier architecture, our approach will be able to operate more efficiently, robustly, and securely within real-world IoT environments.

While LLMs offer exceptional performance and significant advantages, they are not fully reliable, and blind reliance on their outputs can introduce risks. It is therefore essential to evaluate the ethical considerations and privacy implications associated with the use of LLMs in general and within cybersecurity applications in particular.

## 6. Ethic and Privacy

The integration of LLMs into IoT security raises significant ethical considerations that must be addressed to ensure the safety and privacy of users [35]. As LLMs become more prevalent in managing and securing IoT devices, they can inadvertently introduce vulnerabilities that malicious actors might exploit. For instance, the ethical implications of prompt injection and jailbreaking can lead to unauthorized access to sensitive data, compromising user privacy and trust. The exposure of Personally Identifiable Information (PII) through LLM interactions can have severe consequences, as it may lead to identity theft or other forms of exploitation. Moreover, the potential for LLMs to generate or facilitate the dissemination of harmful content, such as hate speech or sexually explicit material, poses ethical dilemmas regarding the responsibility of developers and organizations in mitigating these risks. To address these challenges, it is crucial to develop robust ethical frameworks and evaluative tools that guide the design and deployment of LLMs in IoT environments. Such tools can help ensure that LLMs operate within ethical norms and align with societal values, thereby fostering trust among users. By scrutinizing LLM responses and comparing them to expected human behaviors in moral contexts, developers can better understand the ethical dimensions of their systems and implement necessary safeguards. Ultimately, the ethical use of LLMs in IoT security is not just about preventing misuse; it is about cultivating a responsible approach that prioritizes user safety and privacy while harnessing the benefits of advanced technologies [36]. Additionally, the identification of key features of LLM-based agents can inform the development of strategies to mitigate risks, ensuring that ethical considerations are integrated into the design process.

Furthermore, the growing use of LLMs in sensitive situations raises serious questions about their robustness and adversarial dangers in IoT security applications. Research shows that although while LLMs are being incorporated into a number of applications, there are serious concerns associated with their susceptibility to hostile attacks. LLMs like GPT-3.5 and GPT-4 exhibit varying robustness against adversarial attacks such as misclassification and hallucination, with updates not always ensuring improved security [37]. These attacks can significantly degrade LLM performance, underscoring the need for enhanced safety mechanisms. In the context of IoT systems, especially for intrusion detection, the integration of LLMs presents additional vulnerabilities, as the diverse architectures of IoT networks are particularly susceptible to adversarial exploitation [38]. To address these challenges, strategies like adversarial training and input preprocessing have demonstrated up to a 15% improvement in model accuracy in cybersecurity applications [39]. Furthermore, simplified model architectures such as Gated Linear Attention (GLA) Transformers may offer a practical balance between robustness and efficiency for resource-constrained IoT environments [40]. Despite these risks, LLMs hold significant promise for enhancing IoT security through improved decision-making and anomaly detection, emphasizing the need for ongoing research to develop resilient models capable of adapting to evolving threats.

## 7. Conclusions and Future Work

The integration of LLMs into IoT cybersecurity represents a transformative opportunity to enhance threat detection, automate security policies, and enable intelligent, context-aware defense mechanisms across heterogeneous IoT environments. Prior studies have demonstrated that LLM-based systems can improve anomaly detection, log analysis, and malware classification, particularly when combined with hybrid architectures or privacy-preserving techniques such as federated learning.

In this work, we introduced a practical and efficient Edge-IoT scheme—SEED. Our method leverages a lightweight IoT-level neural network that operates on Edge-generated embeddings, thereby achieving high accuracy of 99.99% with minimal computational and memory requirements on the IoT device. The proposed pipeline enables fine-grained semantic representation of traffic at the Edge level while preserving rapid, low-overhead detection at the IoT edge. Experimental results demonstrated that our EdgeBERT can accurately discriminate among 14 attack classes, while the IoT-level models achieved near-perfect performance in both binary and six-category scenarios. These results highlight the feasibility of integrating transformer knowledge into constrained IoT environments without sacrificing detection performance.

Despite its promising performance, the proposed architecture introduces challenges related to embedding transmission and network reliability. Moreover, embedding vectors may reveal sensitive device information if intercepted, and dependency on edge connectivity can impact inference latency during network disruptions. Addressing these limitations will require secure communication protocols, embedding anonymization mechanisms, and robust fallback strategies to ensure continuous operation.

Future research should explore advanced privacy-preserving embedding techniques, optimized on-device transformer variants, and adaptive edge–IoT load balancing strategies. Further attention should also be given to adversarial robustness, interpretability, and the ethical implications of autonomous security decisions. As IoT deployments continue to expand, the development of lightweight, secure, and resilient LLM-powered defenses will become increasingly essential for protecting large-scale interconnected systems.

## Figures and Tables

**Table 1:** Common Vulnerabilities in IoT Architectures.

IoT Layer	Description	Typical Vulnerabilities	Causes/Challenges	Consequences
Perception Layer	Devices that sense and interact with the physical environment (e.g., sensors, actuators)	Physical tampering, side-channel attacks, firmware manipulation	Low computational power, lack of hardware security, absence of secure boot mechanisms	Unauthorized data access, device malfunction, insertion of rogue devices
Network Layer	Ensures communication between devices and external systems via various protocols	Man-in-the-Middle (MITM), spoofing, sniffing, routing attacks	Use of lightweight protocols with limited encryption, dynamic and decentralized topologies	Data interception or alteration, denial of service, routing manipulation
Application Layer	Interfaces and services exposed to users and external applications	SQL injection, Cross-Site Scripting (XSS), insecure APIs, privilege escalation	Poor input validation, outdated firmware, weak or inconsistent access control policies	Data leakage, unauthorized access, compromised application behavior

**Table 2:** Comparative summary of case studies on LLM applications in IoT cybersecurity, highlighting results and limitations.

Study	Model(s)	Dataset	Key Contribution	Limitations
[10], 2024	SecurityBERT	EdgeIIoTset	98.2%/Privacy-preserving intrusion detection for IoT/IIoT	Lacks real-world deployment analysis; adversarial robustness not evaluated
[18], 2024	BERT, GPT-2	TON-IoT, NSL-KDD, KDDcup99	94.0% (BERT), 91.0% (GPT-2)/IoT malware detection using LLMs	No consideration for deployability; scalability issues
[23], 2024	Federated SecurityBERT	EdgeIIoTset	97.7%/Quantized model for deployment on resource-constrained devices	High communication overhead; delayed model aggregation
[6], 2025	GPT-4 + Autoencoders	KDDcup99 10%	Hybrid AI-based anomaly detection; improved over classical methods	High computational cost; real-time inference feasibility not evaluated
[21], 2025	BART, BERT	CICIoT2023	98.26% (BART), 92.8% (BERT)/Packet classification and intrusion prevention	No discussion on computational demand for IoT deployment
**Ours**	SEED	EdgeIIoTset	Edge-IoT collaborative IDS using tuned BERT (90% reduced model) for embedding generation; 137 KB IoT classifier achieving 99.99%; efficient six-category and binary IoT detection	Relies on stable network connection for embedding querying; edge dependency may impact inference under network instability

**Table 3:** SEED Model Configurations, Parameters, and Memory Sizes.

Model	Architecture	Parameters	Memory Size
EdgeBERT	4 encoder layers, hidden 256, intermediate 512, 4 heads, 2 FC layers (256 × 128, 128 × 15)	10,154,767	40.6 MB
IoT Binary Classifier	2 FC layers (256 × 128, 128 × 1), 1 batch norm (128)	33,281	137 KB
IoT 5-Category Classifier	Same as binary classifier, output 5	33,926	137 KB

**Table 4:** Classification Report for the Edge Model Network.

Class	Precision	Recall	F1-Score	Support
Backd	1.0000	0.9992	0.9996	4972
HTTP	0.9992	1.0000	0.9996	9982
ICMP	1.0000	1.0000	1.0000	23,287
TCP	1.0000	1.0000	1.0000	10,012
UDP	1.0000	1.0000	1.0000	24,314
Finge	0.9746	0.9600	0.9673	200
MITM	1.0000	1.0000	1.0000	243
Norm	1.0000	1.0000	1.0000	96,939
Pass	0.9992	1.0000	0.9996	10,031
Port	0.9998	1.0000	0.9999	4513
Rans	0.9995	0.9982	0.9989	2185
SQL	1.0000	1.0000	1.0000	10,241
Upload	1.0000	1.0000	1.0000	7527
Vul	1.0000	1.0000	0.9996	10,022
XSS	0.9997	1.0000	0.9998	3183

**Table 5:** End-to-End Latency Summary for SEED Inference Pipeline.

Component	Latency
Edge-based model embedding generation	9 ms
IoT-level classifier inference (CPU, 2.5 GHz, 4 cores) *	70 ms
IoT-level classifier inference (NVIDIA T4 GPU)	0.9 ms

* The resource-constrained device for real-world deployment.

## Data Availability

The data used in this study can be found on IEEE DataPort at https://ieee-dataport.org//documents/edge-iiotset-new-comprehensive-realistic-cyber-security-dataset-iot-and-iiot-applications (accessed on 28 October 2025).
